# Photochemical and antioxidative responses of the glume and flag leaf to seasonal senescence in wheat

**DOI:** 10.3389/fpls.2015.00358

**Published:** 2015-05-22

**Authors:** Lingan Kong, Mingze Sun, Yan Xie, Fahong Wang, Zhendong Zhao

**Affiliations:** ^1^Crop Research Institute, Shandong Academy of Agricultural Sciences, Jinan, China; ^2^Plant Protection Station of Liaocheng City, Liaocheng, China

**Keywords:** antioxidative defense, chlorophyll fluorescence, non-photochemical quenching, ultrastructure, wheat, xanthophyll cycle

## Abstract

The non-leaf photosynthetic organs have recently attracted much attention for the breeding and screening of varieties of cereal crops to achieve a high grain yield. However, the glume photosynthetic characteristics and responses to high temperature at the late stages of grain filling are not well known in winter wheat (*Triticum aestivum* L.). In the present study, an experiment was conducted to investigate the anatomy, chloroplast temporal changes, chlorophyll fluorescence, xanthophyll cycle and antioxidative defense system in glumes of field-grown wheat during grain filling compared with flag leaves. Observations using a light microscope revealed that the glumes developed a solid structural base for performing photosynthesis. Compared with the flag leaves, the glumes preserved a more integral ultrastructure, as observed under transmission electron microscopy, and had higher values of Fv/Fm and Φ_PSII_ at the maturity stage. Further analysis of the chlorophyll fluorescence demonstrated that the glumes experienced high non-photochemical quenching (NPQ) at the late stages. Determination of the pool size of the xanthophyll cycle suggested that the (A+Z)/(V+A+Z) ratio was consistently higher in glumes than in flag leaves and that the V+A+Z content was considerably higher in glumes at the maturity stage. In addition, the glumes exhibited a higher antioxidant enzyme activity and a lower accumulation of reactive oxygen species. These results suggest that the glumes are photosynthetically active and senesce later than the flag leaves; the advantages may have been achieved by coordinated contributions of the structural features, higher NPQ levels, greater de-epoxidation of the xanthophyll cycle components and antioxidative defense metabolism.

## Introduction

In wheat, many non-foliar organs, including all parts of the ear and the exposed part of the peduncle, have the ability to assimilate CO_2_ when exposed to light (Wang et al., 2001; Kong et al., 2010). Recently, an increasing body of evidence has proven that the ear has a photosynthetic capacity that is at least comparable to that of the flag leaf (Sanchez-Bragado et al., 2014) and shows a far greater increase of net photosynthesis than flag leaves under elevated CO_2_ (Zhu et al., 2009). Thus, the ear contributes more than the flag leaf to kernel formation under potential yield conditions (Zhou et al., 2014).

The glumes, which are the main photosynthetic organs of the ear, are believed to be a significant source of assimilates for kernel filling in wheat (*Triticum aestivum* L.) and other cereals (Araus et al., 1993b). The photosynthesis of glumes is characterized by recycling of the CO_2_ respired by the developing grains (Gebbing and Schnyder, 2001; Sanchez-Bragado et al., 2014) and by higher ribulose-1,5-bisphosphate carboxylase (RuBPC, EC 4.1.1.39) activity compared with other ear elements (Aliyev, 2012). It appears that ear parts likely have the ability to assimilate CO_2_ through the C_4_ pathway of photosynthesis and to utilize phosphoenolpyruvate carboxylase (PEPC, EC 4.1.1.31) to recapture the respired CO_2_ because, compared with flag leaves, glumes assimilate the fed ^14^CO_2_ and most of the resulting ^14^C in malate but less 3-phosphoglyceric acid under illumination (Singal et al., 1986). These collective results indicate that the glumes actively participate in the process of CO_2_ assimilation during kernel filling.

It has been reported that the glume size is involved in the regulation of grain filling throughout the reproductive stage as both sink and source (Millet and Pinthus, 1984; Lopes et al., 2006). Reserve remobilization from even the topmost leaves to the developing grains may occur indirectly via the glumes in wheat and barley (*Hordeum vulgare* L.; Simpson et al., 1983; Lopes et al., 2006; Feller et al., 2008). During the late stages of grain filling, glumes ensure the transport of a large amount of N compounds to grains (Lopes et al., 2006).

Senescence is a genetically programmed and environmentally regulated developmental process. During grain filling of cereal crops, senescence occurs naturally, involving the coordinated degradation of macromolecules and the reserve remobilization from senescing tissues into reproductive organs (Zimmermann and Zentgraf, 2005). However, as to wheat, a temperate, cool-season C_3_ cereal, high temperature (>27°C) is a common stress at the late grain-filling stages, resulting in the destruction of cellular organelles, premature plant senescence, reductions in the net photosynthetic rate, retardation of grain filling and, consequently, great yield losses (Wardlaw, 2002; Sharma et al., 2008; Kong et al., 2013a). Maximum temperatures of over 35°C occur more commonly across the Chinese wheat belts. Moreover, it has been predicted that the global temperature would increase about 3.5°C in the next 50–75 years and may reach 40°C in many parts of the world’s wheat growing areas during grain filling, and then exerts a severe heat stress to the moderate-climate crop (Kong et al., 2013a).

Photosynthesis is heat-sensitive and this physiological process is significantly inhibited by high temperatures (Wang et al., 2015). in addition to high photochemical light-use efficiency that is commonly monitored using chlorophyll fluorescence, the heat dissipation through the xanthophylls cycle and non-photochemical quenching (NPQ) of chlorophyll fluorescence play an important role in protecting plants from stresses and thus correlate with the resistance to senescence (Björkman and Demmig-Adams, 1995; Demmig-Adams and Adams, 1996; Wang et al., 2015).

Photosynthesis is performed in chloroplasts and regulated by many factors such as anatomy and chloroplast ultrastructure of green tissues. And stomata play a vital role in regulating plant photosynthetic capability as a main CO_2_ diffusion pathway (Singh et al., 2013; Duan et al., 2015). As green tissues progress toward senescence, photosynthetic rate would decrease due to the ultrastructural alterations of chloroplasts (Kong et al., 2010) and the ROS levels would increase due to a gradual decline in antioxidant protection (Zimmermann and Zentgraf, 2005). Moreover, the high temperatures combined with strong solar irradiation that occur during the midday hours may result in the ROS production (Buchner et al., 2015). In plant, the main enzymatic ROS scavengers include superoxide dismutases (SOD), catalases (CAT), and various peroxidases are the primary antioxidant enzymes (Zimmermann and Zentgraf, 2005; Wang et al., 2015).

Glumes may have a higher ability to resist abiotic stress than other organs. In bread wheat, for example, the glumes maintain a higher relative water content under progressive water stress than the flag leaves (Wardlaw, 2002). Under dry conditions, glumes exhibit higher water use efficiency (WUE) than leaves, contributing significantly to grain filling due to their photosynthetic activity in refixing respiratory CO_2_ (Bort et al., 1996). Cultivars with a relatively large ear (glume) area are advantageous for intercepting a greater proportion of incident light during grain filling and show high WUE (Bort et al., 1996). It would appear that the glumes exhibit a slower rate of senescence at the late grain-filling phases than flag leaves (Bort et al., 1996; Lopes et al., 2006) and that glumes may thus play a more prominent role at the late stages.

However, compared with green leaves, the photosynthetic characteristics and mechanism of senescence resistance in glumes are not well understood. Although several studies have analyzed the role of glumes from a photosynthetic viewpoint, its potential contribution to grain development is not clear. The aims of this study were to compare the photosynthetic characteristics of glumes in winter wheat with those of flag leaves, which were used as a control, and to evaluate the mechanism through which glumes resist senescence during the late phase of grain filling.

## Materials and Methods

### Plant Materials and Growth Conditions

Winter wheat (*Triticum aestivum* L.) of the cultivar Jimai 22 was planted in 2012–2013 in a field at an experimental station (36°42′ N, 117°4′ E; altitude 48 m) of the Shandong Academy of Agricultural Sciences, China. Jimai 22 is a high-yield cultivar that is most popularly planted in the Yellow River and Huai River Valleys of China. The glumes of this cultivar, particularly at the basal part, generally senesce later than the flag leaves. Seeds of Jimai 22 were sown on 8 October, 2012 at a rate of 15 g m^–2^. The climate in this region is continental and warm with an average annual temperature of 13.6°C and an average rainfall of ∼660 mm. The soil type is classified as sandy loam (pH 7.4). The top 40 cm of soil contained 67.3 mg kg^–1^ water-hydrolysable nitrogen, 22.1 mg kg^–1^ rapidly available phosphorous, 139.3 mg kg^–1^ rapidly available potassium and 2.05% organic matter.

**FIGURE 1:**
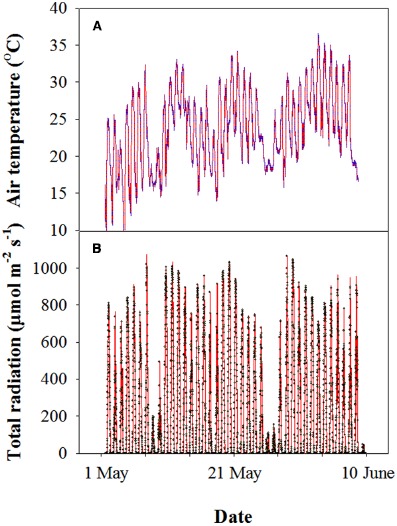
**Representative profile of the air temperature (A) and photosynthetically active photon flux density (PPFD) (B) of wheat during grain filling in the 2012–2013 cropping year at the experimental site.** Each datum was collected every 10 min using a mini-type weather station.

Before sowing, 11 g m^–2^ N, 15 g m^–2^ P_2_O_5_, and 15 g m^–2^ K_2_O were applied to the soil. At the shooting stage, 11 g m^–2^ N of urea was top-dressed. All of the analyses were conducted from 3 May to 4 June, 2013. During this period, the air temperature was between 11 and 36°C, and the average photosynthetic photon flux density (PPFD) was approximately 1000 μmol m^–2^ s^–1^ at midday (Figure 1).

### Stomatal Density

The stomatal density on the glumes and flag leaves was measured. The organs were coated with a thick layer of nail polish, and the dried polish was carefully peeled off the organ and placed on a microscope slide to count the number of stomata (Teare et al., 1971). The stomatal of both sides of the flag leaves (abaxial and adaxial) and glumes (external and internal) were randomly counted in optical microscope (Zeiss Axioskop 40, Leica, Germany). The number of stomata and cell are presented on area basis as stomatal density (mm^2^). Each value represents the mean ± standard deviation (SD) from five ears. At least four glumes were measured in each ear and the results were averaged to give a single value for each ear.

### Anatomy and Ultrastructure

The flag leaves and glumes were sampled 0, 8, 16, 24, and 32 days after anthesis (DAA). Cross-sections with a width of 1-2 mm were immediately fixed in 2.5% (v/v) glutaraldehyde solution in 100 mM sodium phosphate buffer (SPB) (pH 7.2) at 4°C for 24 h. The samples were subsequently rinsed in SPB, dehydrated through a graded ethanol series, transferred into propylene oxide and embedded in epoxy resin (Epon812, Shell Chemical, Houston, TX, USA). Four 2-μm-thick sections were cut from each sample using an LKB-V microtome. The sections were mounted on microscope slides and stained with toluidine blue O. The anatomical structure was observed and photographed using a light microscope (Zeiss Axioskop 40, Leica, Germany) equipped with a charge-coupled device (CCD) camera. Ultrathin sections (60 nm) were cut with a LKB-V microtome and stained with 2% (w/v) uranyl acetate in 70% (v/v) methanol and 0.5% (w/v) lead citrate. The ultrastructure was observed with a JEM-1230 transmission electron microscope (TEM; JEOL Ltd., Tokyo, Japan) at 80 kV.

### Chlorophyll Fluorescence Assay And Imaging

Chlorophyll fluorescence measurements were performed at 8, 16, 24, and 32 DAA to determine the maximum PSII quantum yield (Fv/Fm), effective PSII quantum yield (Φ_PSII_) and NPQ in the adaxial sides of the flag leaves and dorsal sides of the glumes. After a dark acclimation period of 30 min, the whole leaf or glume was measured using a kinetic imaging fluorometer to determine the chlorophyll fluorescence parameters (Fluor-Cam, Photon System Instruments Ltd., Brno, Czech Republic) according to Nedbal et al. (2000). The duration of Fo (the minimum fluorescence in the dark-adapted state) measurement was 5.04 s. After measuring Fo, the samples were illuminated with a saturating pulse (1500 μE m^2^ s^–1^) to determine the maximal fluorescence in the dark-adapted state (Fm). The samples were then illuminated with actinic light, and 0.7-s saturating flashes were applied. The maximum fluorescence of the light-adapted samples (Fm′) and the steady-state fluorescence (Fs) were recorded. Chlorophyll fluorescence emission transients were captured through a series of images with a resolution of 512 × 512 pixels using a CCD camera. Numerical analyses of the classical physiological parameters were performed as follows: maximum PSII quantum yield as Fv/Fm = (Fm–Fo)/Fm, quantum efficiency of PSII electron transport as Φ_PSII_ = (Fm′–Fs)/Fm′ and the non-photochemical chlorophyll fluorescence quenching parameter as NPQ = (Fm–Fm′Fm′).

### Pigment Analyses

Flag leaf and glume samples were collected and ground to a fine powder in liquid N_2_. The pigments were then extracted in cold 95% acetone at 4°C for 24 h. The resulting extracts were filtered through a 0.45-μm membrane filter. The pigments were separated and quantified by HPLC using a C_30_ column (250 × 4.6 mm, 5 μm; YMC Europe, Schermbeck, Germany) as described previously (Takeda et al., 1996).

### Assays of Antioxidant Enzyme Activity

Assays of the antioxidant enzyme activities were measured as described by Kong et al. (2013b,c). The flag leaves and glumes were harvested at 0, 8, 16, 24, and 32 DAA, immediately frozen in liquid N_2_, and then stored at –80°C until experimental analysis. Frozen samples (*ca*. 0.5 g) were ground to a fine powder using a mortar and pestle under liquid N_2_. The soluble proteins were extracted by homogenizing the powder in 10 ml of 50 mM SPB (pH 7.0) containing 1 mM EDTA and 1% polyvinylpyrrolidone (PVP-40). The homogenate was centrifuged at 12,000 × g and 4°C for 20 min, and the supernatant was used to determine the activities of peroxidase (POD, EC 1.11.1.7), catalase (CAT, EC 1.11.1.6), and superoxide dismutase (SOD, EC 1.15.1.1). The water-soluble proteins were determined using the Bradford (1976) assay (Bradford, 1976). The data were averaged from three replicates.

### Determination of Reactive Oxygen Species

The ROS concentration was determined using 2′,7′-Dichlorofluorescein diacetate (DCFH-DA) (Sigma-Aldrich, Germany) (which is oxidized by ROS to DCF) as described by Kong et al. (2013b) with slight modifications. A 25 mM solution of DCFH-DA was prepared in dimethyl sulfoxide and maintained at –20°C until use. Sections cut from the flag leaves or the whole glumes (*ca.* 0.2 g) were washed in 50 mM methyl ethanesulfonate buffer (pH 6.2) and transferred to 100 μl of fresh buffer in small wells of ELISA plates containing 10 μM DCFH-DA. Following incubation at 25°C in the dark for 20 min, the fluorescence was immediately measured with an excitation wavelength of 485 nm and an emission wavelength of 535 nm using an ELISA plate reader (GENios Pro, Tecan, Switzerland). The ROS concentration units were defined as the average increase in the DCF florescence per g of fresh sample per minute.

### Statistical Analysis

The statistical analyses were performed using the data processing system (DPS) statistical software (v.14.10, Refine Information Tech. Co., Ltd., Hangzhou, Zhejiang, China). Duncan’s multiple range test was used to evaluate the statistical significance of the results obtained. The data are presented as the means ± SD, and significant differences among the treatments are shown by different letters. A general linear model ANOVA was used for statistical analysis of mixed effects of the organ (glume and flag leaf) and the date of sampling on chlorophyll fluorescence, antioxidative enzymes, ROS concentration and xanthophyll cycle.

## Results

### Anatomy and Stomatal Density

**FIGURE 2:**
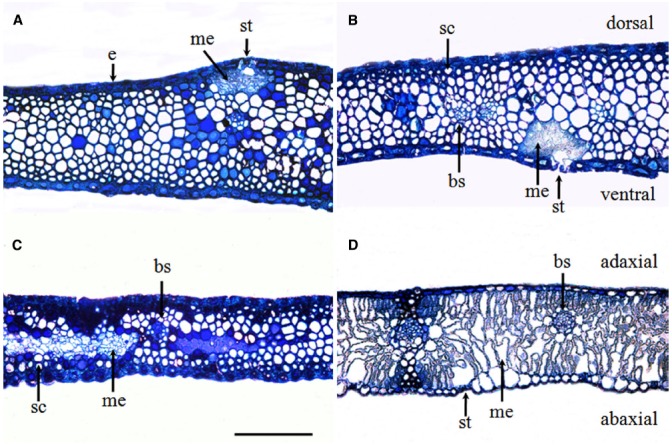
**Glume (A–C) and flag leaf (D) cross-sections were stained with toluidine blue O to show the anatomy at the anthesis of wheat cv. Jimai 22.** Note the chlorenchyma strands and the localization of mesophyll cells in the center of glumes. Abbreviations: ab, abaxial side; ad, adaxial side; bs, bundle sheath; cs, chlorenchyma strands; d, dorsal; e, epidermis; me, mesophyll cell; sc, sclerenchyma cell; and v, ventral (bar = 100 μm).

The glume of winter wheat presented a thick epidermis on both the dorsal and ventral sides and sub-epidermal sclerenchymatous tissue with thick walls (Figures 2A,B). In comparison with the flag leaves (Figure 2D), the glumes developed more sclerenchyma cells and fewer mesophyll cells (Figure 2C). The chlorenchyma strands were localized toward both the adaxial and the abaxial sides, composed of cells rich in chloroplasts at the anthesis and accompanied by the presence of stomata (Figures 2A,B), whereas the flag leaves showed a relatively even distribution of mesophyll cells across the cross section (Figure 2D).

Compared with the flag leaves, the glumes showed an asymmetric distribution of stomata on each side of the organ. Although stomata are present on the ventral side (i.e., facing the grains), glumes have a higher stomatal frequency on the dorsal side (i.e., external). The average stomatal density reached 63.23 ± 4.89 mm^–2^ on the dorsal side and 80.56 ± 9.16 mm^–2^ on the ventral side of the glumes. This measure was significantly higher than the density measured on the adaxial face of the flag leaves and the density recorded on the abaxial face of the flag leaves (Table 1).

**TABLE 1:** **Stomatal density of the flag leaf and glume in wheat**.

**Organ**		**Number of stomata (mm^–2^)**
Flag leaf	Adaxial	57.83 ± 6.56 b
	Abaxial	44.75 ± 6.37 c
Glume	Dorsal	63.23 ± 4.89 b
	Ventral	80.56 ± 9.16 a

Each value represents the mean ± SD of five ears and at least four glumes were measured in each ear. Means followed by different letters differed significantly at P < 0.05 according to Duncan’s multiple range test.

### Ultrastructure

**FIGURE 3:**
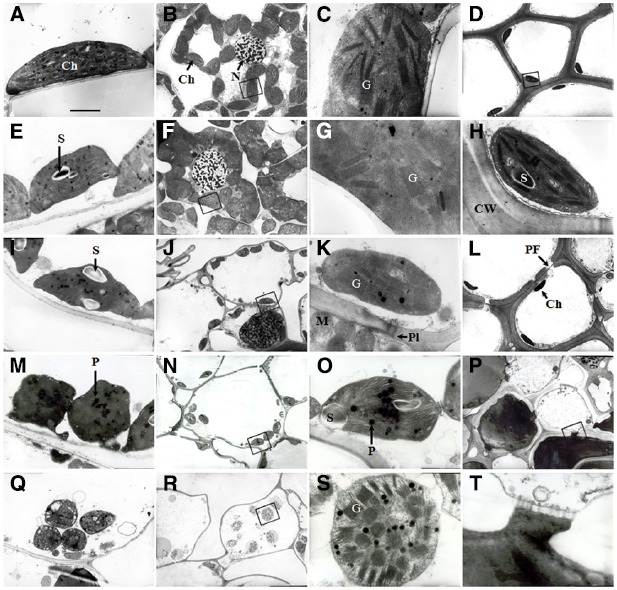
**Transmission electron micrographs showing the ultrastructure of cells in wheat flag leaves (A, E, I, M, and Q) and glumes (B–D, F–H, J–L, N, O, P, R, S, and T) at 0, 8, 16, 24, and 32 DAA of wheat. (A–D** and **H)**: 0 DAA; **(E–G)**: 8 DAA; **(I–L)**: 16 DAA, **(M–P** and **T)**: 24 DAA; **(M–P** and **T)**: 24 DAA. **(Q–S)**: 32 DAA. **(C, G, H, K, O, S)**, and **(T)**: higher magnification of the areas highlighted in **(B, F, D, J, N, R)**, and **(T)**, respectively. Abbreviations: Ch, chloroplast; G, granum; PF, pit field; Pl, plasmodesma; P, plastoglobule; S, starch grain. Scale bars: 500 nM **(A, E, I, M, P** and **Q)**, 2 μM **(B, D, F, J, L, N** and **R)**, and 50 nM **(C, G, K, O, S** and **Q)**.

Transmission electron micrographs obtained from the flag leaves during the initial stages of (0–8 DAA) showed that the cells of the flag leaves had well-differentiated chloroplasts containing fully developed grana with numerous layers and well-developed stroma lamellae with several starch granules (Figures 3A,E). As grain-filling progressed (16 DAA), the chloroplasts were characterized by an irregular arrangement of the thylakoid stacks, an apparently declining amount of starch and a marked increase in the number of plastoglobuli, indicating the commencement of total chloroplast degradation and leaf senescence (Figure 3I). The most striking feature at this stage was the frequently observed plasmodesmata between parenchyma cells and pit fields in the wall of sclerenchyma cells (Figures 3K,L). At 24 DAA, the chloroplast shape changed from ellipsoidal to pronouncedly more spherical. Marked disorganization of thylakoid membranes together with the accumulation of plastoglobuli was also observed (Figure 3M). At the maturity stage (32 DAA), the entire structure of the chloroplasts ruptured, and the thylakoid membranes were nearly completely disintegrated (Figure 3Q).

The glumes exhibited a lack of organelles and the thickening of cell walls in the cells of tissues other than the mesophyll. At 0 and 8 DAA, the mesophyll cells in the chlorenchyma strands (Figures 2A,B) contained a large number of well-differentiated chloroplasts, and in some cases, the chloroplasts huddled together (Figures 3B,F). The chloroplasts in both the chlorenchyma cells (Figures 3C,G) and the sclerenchyma cells (Figures 3D,H) had fully developed grana with many layers of well-developed grana lamellae but fewer starch granules than in the flag leaves. At 16 DAA, even though the number of chloroplasts decreased and the nuclei in some cells disappeared, the chloroplasts in the glumes contained more thylakoids than those in the flag leaves (Figures 3J,K). Additionally, the chloroplasts in the glume mesophyll cells accumulated fewer plastoglobuli compared with the flag leaves. This observation indicates that the overall degradation of chloroplast components was markedly slower because the plastoglobuli were thought to contain material from disintegrated thylakoid membranes (Burke et al., 1984). At 24 DAA, an apparent increase in the plastoglobule content was observed. Although the parallel arrangement of the grana lamellae was lost in some chloroplasts and some of the thylakoids became swollen, starch inclusions still occurred in the chloroplasts (Figures 3N,O), indicating that photosynthesis was still active at this stage. At 32 DAA, the number of chloroplasts decreased noticeably, and the envelope was ruptured. However, the grana lamellae were still abundant in comparison to the complete disruption of the thylakoid membrane system in the flag leaves (Figures 3R,S). At the maturity stage, a redistribution of phenols occurred between sclerenchyma cells (Figures 3P,T).

### Chlorophyll Fluorescence

**FIGURE 4:**
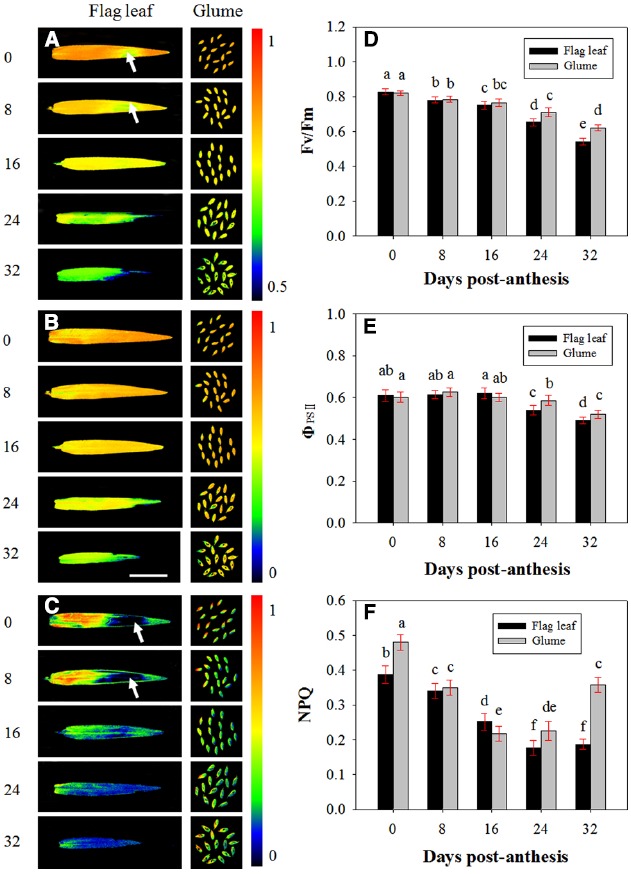
**Fluorescence imaging of the maximal efficiency of PSII photochemistry (Fv/Fm, A, D), actual PSII efficiency (Φ_PSII_, B, E) and non-photochemical quenching (NPQ, C, F) of flag leaves and glumes in wheat (*Triticum aestivum* var. Jimai 22) during grain filling.** The measurements were performed at 0, 8, 16, 24, and 32 DAA. The data were determined in the year 2013. Each value is the mean ± SD from at least six leaves or 30 glumes. The columns labeled with different letters differed significantly at *P* < 0.05 according to Duncan’s multiple range test using DPS software.

The PSII photochemistry in the flag leaves and glumes in the dark-adapted state was investigated during grain filling. Figure 4 demonstrates the changes in Fv/Fm (Figures 4A,D), Φ_PSII_ (Figures 4B,E) and NPQ (Figures 4C,F) in the flag leaves and glumes during grain filling. After anthesis, the values of Fv/Fm and Φ_PSII_ in the flag leaves and glumes gradually decreased, and no difference was observed between both organs at 0, 8, and 16 DAA. In subsequent stages, the decline was much greater in flag leaves than in glumes. As a result, the values of Fv/Fm and Φ_PSII_ in the glumes were significantly higher at 24 and 32 DAA compared with those found for the flag leaves (*P* < 0.05). In addition, the differences in Fv/Fm and Φ_PSII_ between both organs were significant during the grain filling phrase (Table 2).

**TABLE 2:** **Statistical analysis of effects of the organ (glume and flag leaf) and the date of sampling on chlorophyll fluorescence, antioxidative enzymes, ROS concentration, and xanthophyll cycle**.

**Source of variation (degrees of freedom)**	**Organ (1); *F*, *P***	**Date of sampling (4); *F*, *P***	**Organ × date of sampling (4); *F*, *P***
Fv/Fm	29.37, < 0.01	244.33, < 0.01	8.65, < 0.01
Φ_PSII_	4.11, 0.05	44.55, < 0.01	3.53, 0.01
NPQ	82.81, < 0.01	173.09, < 0.01	31.04, < 0.01
V+A+Z	233.11, < 0.01	35.57, < 0.01	43.92, < 0.01
(A+Z)/(V+A+Z)	35.03, < 0.01	133.88, < 0.01	0.78, 0.55
SOD	128.34, < 0.01	65.14, < 0.01	47.08, < 0.01
POD	811058, < 0.01	144.63, < 0.01	102.11, < 0.01
CAT	5.10, 0.03	415.45, < 0.01	59.43, < 0.01
ROS concentration	16.13, < 0.01	45.22, < 0.01	2.26, 0.08

Contrary to our anticipations, the highest values of NPQ were observed at 0 DAA in both organs. Additionally, the value of NPQ in the glumes was significantly higher than that in the flag leaves (0.387 *vs.* 0.480). At 8 and 16 DAA, the NPQ in the glumes decreased more sharply than that in the flag leaf, and the glumes thus had a similar NPQ value to that found in the flag leaves at 8 DAA and a significantly lower value than that obtained in the flag leaves at 16 DAA. However, at 24 and 32 DAA, this value in the glumes was increased and was significantly higher than that in the flag leaves. Similarly to Fv/Fm and Φ_PSII_, the differences in NPQ values between both organs were significant during the grain filling phrase (Table 2). In addition, we found that the heterogeneous variability in NPQ across the leaf area was in accordance with that found for Fv/Fm: in the regions with a relatively high Fv/Fm, the NPQ was increased, whereas in the regions with a low Fv/Fm ratio, the NPQ values were also low (Figures 4A,C).

### Xanthophyll Cycle

**FIGURE 5:**
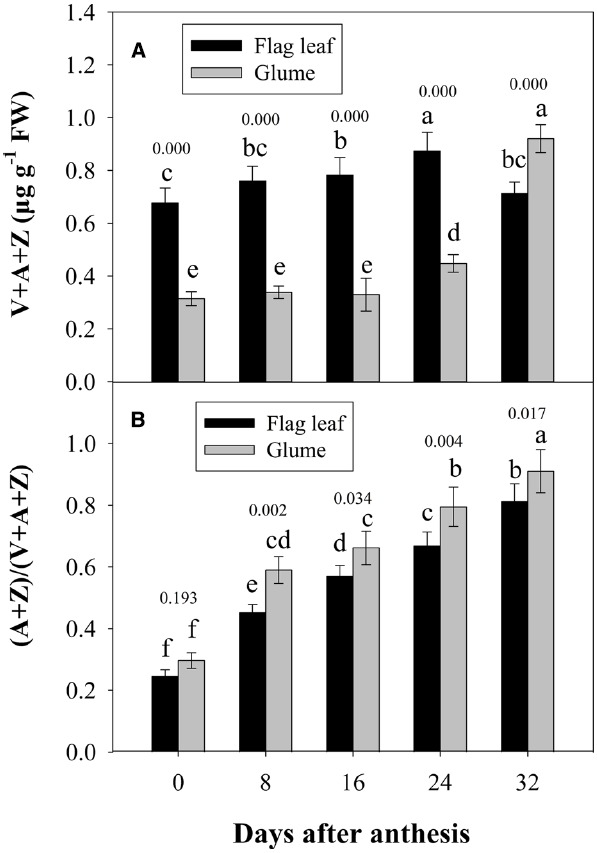
**Changes in the xanthophyll cycle pool size, i.e., contents of V+A+Z (A), and the de-epoxidation state of the xanthophyll cycle, i.e., the (A+Z)/(V+A+Z) ratio (B).** Columns with different letters are significantly different according to Duncan’s multiple range test using DPS software (*P* < 0.05). The *P* value for comparison between flag leaf and glume at each stage is presented over the top of column. The values are the means ± SD from three independent measurements.

Figure 5 shows the changes in the pool size of the xanthophyll cycle, i.e., violaxanthin (V) + zeaxanthin (Z) + antheraxanthin (A), and the conversion state of the xanthophyll cycle, i.e., the ratio of (A+Z) to (V+A+Z), in the flag leaves and glumes during grain filling. The pool size of the xanthophyll cycle in the glumes was significantly lower than that in the flag leaves at 0, 8, 16, and 24 DAA. However, contrary to the marked decreases observed in the flag leaves, the overall content of xanthophylls was significantly increased in the glumes at 32 DAA and was thus significantly higher in the glumes than in the flag leaves (Figure 5A). The extent of the de-epoxidation of the pigment interconversion within the xanthophyll cycle can be described by the (Z+A)/(V+Z+A) ratio. This ratio increased continually in both tissues during grain filling as the air temperature continually increased (Figures 5B and 2S). In glumes, the (Z+A)/(V+Z+A) ratio was consistently and significantly higher than that in the flag leaves (Figure 5B; Table 2).

### Antioxidative Defense and Ros Relative Concentration

**FIGURE 6:**
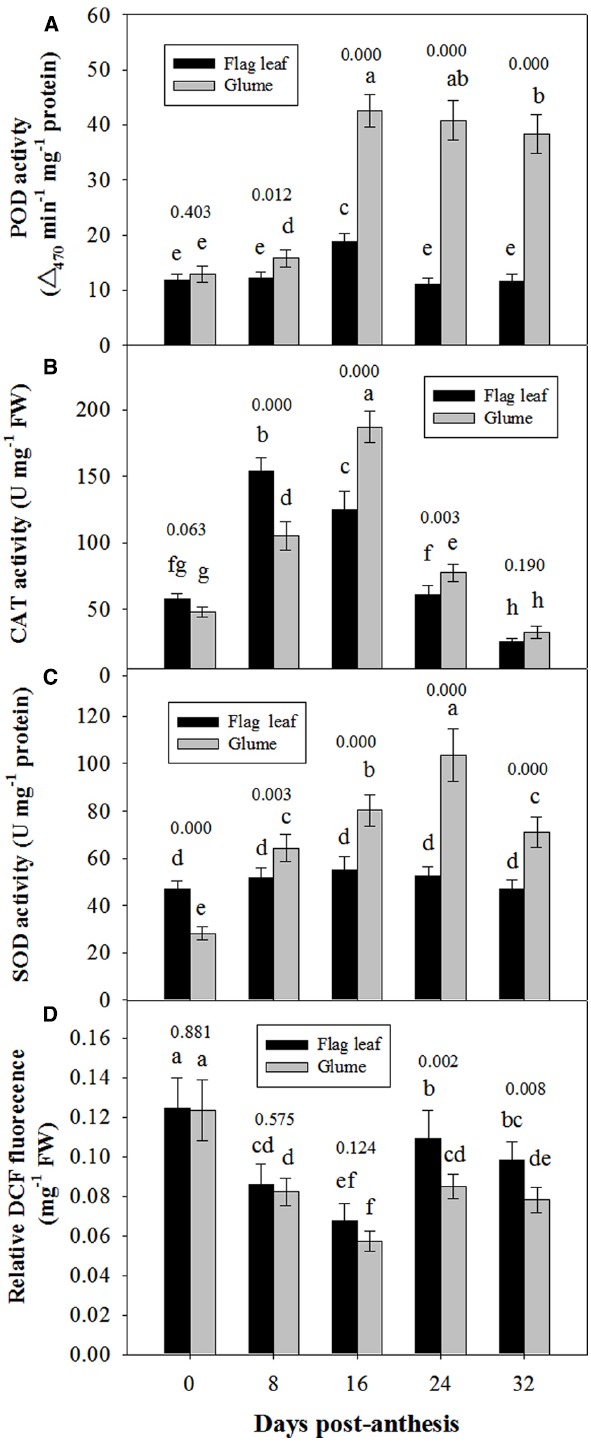
**Comparison of the activities of POD (A), CAT (B) and SOD (C) and of the relative DCFH-DA fluorescence intensity between the glumes and flag leaves (D).** The ROS concentration was measured using DCFH-DA, which is oxidized by ROS to DCF. The fluorescence was determined at 20 min after the incubation of glumes or flag leaf cross-sections with DCFH-DA. Each value represents the mean ± SD from four independent samples. The columns labeled with different letters are significantly different at *P* < 0.05 according to Duncan’s multiple range test using DPS software. The *P* value for comparison between flag leaf and glume at each stage is presented over the top of column.

Figure 6 shows the changes in the activities of POD, CAT and SOD in the glumes and flag leaves. At 0 DAA, the POD activity of the glumes was similar to that of the flag leaves (Figure 6A); whereas the CAT and SOD activities of the glumes were lower than those of the flag leaves (Figures 6B,C). At 8 DAA, the activity of these enzymes increased to different extents in both organs; therefore, the glumes presented higher POD and SOD activity and lower CAT activity. At subsequent stages, the glumes showed consistently higher activities of these enzymes, particularly POD (Figure 6). Effects of the organ (glume and flag leaf) and the date of sampling on the activities of these antioxidative enzymes was significant (Table 2).

The ROS relative concentration was evaluated by measuring the fluorescence arising from the oxidation of DCFH-DA that occurred in both glumes and flag leaves. At 0 and 8 DAA, the glumes showed a similar concentration to the flag leaves. However, at 16, 24, and 32 DAA, we found that the ROS relative concentration was constantly lower in the glumes than in the flag leaves (Figure 6D), although the organ × date of sampling interaction effect on ROS concentration was not significant (Table 2).

## Discussion

It has been suggested that photosynthesis ear accounts for a high grain yield. In wheat, the photosynthesis in glumes and awns can provide up to 30% of the total grain carbon (Parry et al., 2011). Moreover, in terms of anatomical features, the glumes may have an intermediate C_3_–C_4_ pathway (Ziegler-Jöns, 1989) and a significantly higher activity ratio of PEPC to Rubisco compared with the flag leaves (Xu et al., 2004). In the present study, we found that glumes had mesophyll cells containing chloroplasts and that both the ventral and dorsal sides presented stomata, indicating that the glumes may be photosynthetically active. These observations that the stomata is positioned on the internal side (i.e., facing the grains) and that chloroplasts are present in the sclerenchymatous cells of glumes (Figures 6A, 2D,H, and L) strongly support the findings reported by Araus et al. (1993a,b), Gebbing and Schnyder (2001) and Sanchez-Bragado et al. (2014), who have showed that glumes can re-fix CO_2_ released from respiration in the ear organs (Araus et al., 1993a,b; Gebbing and Schnyder, 2001; Sanchez-Bragado et al., 2014). Similar results were observed by Steinmeyer et al. (2013).

Photosynthetically active organs are characterized by well-developed chloroplasts with a high proportion of grana stacks and stromal thylakoids (Kong et al., 2010). The dilatation of the thylakoids, the degradation of the membrane system and the disintegration of cell organelles, particularly chloroplasts, are important indicators of the extent of senescence (Kong et al., 2010; Farooq et al., 2011; Kang et al., 2015). In the present study, transmission electron micrographs showed that chloroplasts in both flag leaf and glume during the initial stages of anthesis contained fully developed grana, well-developed stroma lamellae with several starch granules and a small number of plastoglobuli (Figures 3A–H). As grain-filling progressed, the thylakoid membranes of chloroplasts in both organs began to swollen, with an irregular arrangement of thylakoid stacks, the declined amount of starch, the markedly increased number of plastoglobuli (Figures 3I,K,M,O), indicating the onset of plant senescence. However, at the late stages of grain filling, the chloroplasts in the glumes remained more structurally preserved at 24 and 32 DAA (Figures 3Q,S). This finding is in accordance with the field observation that the glume of cultivar Jimai 22 yellowed late compared with the flag leaves, indicating the glumes showed delayed senescence.

Under heat stress, senescence is accelerated due to an inhibition of chlorophyll biosynthesis and the accelerated breakdown of thylakoid components (Farooq et al., 2011). Loss of photosynthetic competence is therefore considered one of the key indicators of the senescence and decrease in PSII activity of photosynthetic organs (Lu et al., 2002; Kong et al., 2010). Given that the integrity of chloroplasts and well-conserved thylakoid membranes are essential for photosynthesis, a close relationship is expected to exist between the photosynthetic rate and the chloroplast ultrastructure during grain development. In the present study, we characterized the PSII photochemistry of glumes and found that the Fv/Fm and Φ_PSII_ values of PSII photochemistry were higher in glumes than flag leaves at the late stages of grain filling, suggesting that the glumes exhibited a higher photosynthetic efficiency (Figures 4A,B,D,E; Table 2). Therefore, it is reasonable to propose that the glumes senesced later than the flag leaves of wheat Jimai 22 at the late stages.

At the late stages, wheat is often exposed to high temperature and high light conditions (Figure 1). And glumes are more sun-exposed, receive more light energy and may be subjected to higher ambient temperature compared with flag leaves. Normally, photosynthesizing organs cannot utilize all of the light absorbed during exposure to full sunlight for photosynthesis (Björkman and Demmig-Adams, 1995; Demmig-Adams and Adams, 1996). Therefore, an imbalance between energy absorption and photosynthetic utilization may be expected. Thus, there is a need for photoprotection under the stress of excess light and high temperature. NPQ is a mechanism that protects photosynthetic organisms against excessive irradiation and is commonly used to estimate the rate constant for heat loss from PSII (Kotabová et al., 2011; Murchie and Lawson, 2013). To investigate the mechanism of higher resistance of glumes to the senescence compared with flag leaves, we further analyzed the NPQ efficiency. We found that the values of NPQ were higher in glumes than in flag leaves at 24 and 32 DAA (Figures 4C,F) in the late stages of grain filling with higher air temperature, indicating that the glumes allocated more energy to NPQ pathways. Thus, the glumes preserved greater thermal dissipation in the warm climate during the late stages, which is associated with higher temperature conditions than the optimum. Interestingly, the regional accordance between NPQ and Fv/Fm across the leaf area indicates that the NPQ is closely associated with the quantum efficiency of PSII photochemistry.

The xanthophyll cycle has been reported to regulate thermal dissipation in the light harvesting complex and the synergetic effects of NPQ, and the involvement of the xanthophyll cycle in the protection of photodamage to plants has also been observed in *Chromera velia*, *Oryza sativa* and *Brassica campestris,* but the exact mechanism of action remains to be elucidated (Kotabová et al., 2011; Zhu et al., 2011). The xanthophyll cycle, which occurs in the thylakoid membranes of all higher plants, is composed of carotenoid pigment interconversions from V to Z and A. It is widely accepted that the xanthophyll cycle involves the dissipation of excess excitation energy in the antennae complexes of PSII as heat. Under conditions of excess excitation energy, the xanthophyll cycle pigments are thought to be involved in the photoprotective dissipation process (Demmig-Adams and Adams, 1992). We therefore compared the changes in the xanthophyll cycle pigments in the glumes and flag leaves during grain filling to evaluate the capacity of both organs for photoprotective dissipation of excess excitation. We observed that there was a greater proportion of the xanthophyll cycle pool at maturity (Figure 5A) and a greater increase in the ratio of (V+A) to (V+A+Z) during grain filling in the glumes than in the flag leaves (Figure 5B; Table 2). An increase in this ratio has been thought to be an energy dissipation mechanism that protects the photosynthetic apparatus against excess light (Demmig-Adams and Adams, 1992). We argue that the interconversion efficiency of V to A and Z in glumes is higher than that in flag leaves; thus, the xanthophyll cycle plays an important role in alleviating photoinhibition in the glumes by dissipating more excess energy during exposure to high light and temperature stress.

During plant senescence, the integrity and photosynthetic ability of PSII are closely associated with the levels of ROS; In particular, abiotic stresses, such as drought, heat and overexcitation, can lead to an excessive accumulation of ROS and consequently to photo-oxidative damage to the photosynthetic apparatus and cellular macromolecules, which can then lead to senescence (Apel and Hirt, 2004; Vlčková et al., 2006; Chen et al., 2011). In our study, we investigated the capacity of the antioxidative defense system to evaluate its role in resisting senescence during grain filling. We observed that the glumes presented higher antioxidative enzyme activities (particularly POD activity) and lower accumulation of ROS at the late stages (Figure 6). Because high activities of antioxidant enzymes are closely associated with the delayed appearance of senescence and a higher net photosynthesis under stress conditions (Kreslavski et al., 2009; Huseynova, 2012), we can reasonably postulate that the enhanced antioxidant activities observed may contribute to the delayed senescence, thermo-resistance and higher photosynthetic capacity of the glumes compared with the flag leaves.

In addition to the enzymatic ROS scavenging system, phenolic compounds are considered a type of strong non-enzymatic antioxidant due to the availability of phenolic hydrogen (Sharma et al., 2012; Taheri et al., 2014). In the present study, a high level of phenolics was accumulated in the cells of glumes but not of flag leaves (Figures 3P,T), indicating that the oxidation of phenolics by ROS may be a potential way to scavenge ROS.

In conclusion, wheat glumes have a solid anatomical base for photosynthesis, exhibit a higher efficiency of energy dissipation by the NPQ pathway and xanthophyll cycle and present a greater antioxidant competence, and all of these processes may contribute to a greater integrity of thylakoid membrane systems, heat stability, delayed senescence and higher photosynthetic capability of glumes at the late stages of grain filling. Therefore, the glumes may play a substantial role in contributing to grain filling and may be even more important than the flag leaves. This situation may have interesting consequences regarding the attention that breeders should pay to ears when designing new ideotypes.

### Conflict of Interest Statement

The authors declare that the research was conducted in the absence of any commercial or financial relationships that could be construed as a potential conflict of interest.

## Abbreviations

A: antheraxanthin
CAT: catalase
Fm and Fm’: maximal fluorescence in dark- and light-adapted leaves, respectively
Fo: minimal fluorescence in dark-adapted samples
Fs: steady-state fluorescence
Fv: maximum variable fluorescence in dark-adapted samples
Fv/Fm: maximum efficiency of PSII photochemistry
NPQ: non-photochemical quenching
PEPC: phosphoenolpyruvate carboxylase
POD: peroxidase
PSII: photosystem II
ROS: reactive oxygen species
RuBPC: ribulose-1,5-bisphosphate carboxylase
SOD: superoxide dismutase
V: violaxanthin
Z: zeaxanthin
Φ_PSII_: Light-adapted actual photochemical yield of PSII.
